# Mathematical Ability and Socio-Economic Background: IRT Modeling to Estimate Genotype by Environment Interaction

**DOI:** 10.1017/thg.2017.59

**Published:** 2017-11-06

**Authors:** Inga Schwabe, Dorret I. Boomsma, Stéphanie M. van den Berg

**Affiliations:** 1 Department of Research Methodology, Measurement and Data Analysis (OMD), University of Twente, Enschede, the Netherlands; 2 Department of Methodology and Statistics, Tilburg University, Tilburg, the Netherlands; 3 Department of Biological Psychology, Vrije Universiteit, Amsterdam, the Netherlands

**Keywords:** mathematical ability, SES, IRT, genotype by environment interaction

## Abstract

Genotype by environment interaction in behavioral traits may be assessed by estimating the proportion of variance that is explained by genetic and environmental influences *conditional* on a measured moderating variable, such as a known environmental exposure. Behavioral traits of interest are often measured by questionnaires and analyzed as sum scores on the items. However, statistical results on genotype by environment interaction based on sum scores can be biased due to the properties of a scale. This article presents a method that makes it possible to analyze the actually observed (phenotypic) item data rather than a sum score by simultaneously estimating the genetic model and an item response theory (IRT) model. In the proposed model, the estimation of genotype by environment interaction is based on an alternative parametrization that is uniquely identified and therefore to be preferred over standard parametrizations. A simulation study shows good performance of our method compared to analyzing sum scores in terms of bias. Next, we analyzed data of 2,110 12-year-old Dutch twin pairs on mathematical ability. Genetic models were evaluated and genetic and environmental variance components estimated as a function of a family's socio-economic status (SES). Results suggested that common environmental influences are less important in creating individual differences in mathematical ability in families with a high SES than in creating individual differences in mathematical ability in twin pairs with a low or average SES.

In the classical twin study, often phenotypic variance in a trait, σ^2^_*P*_, is decomposed into variance due to additive genetic (A) influences (σ^2^_A_), common-environmental (C) influences (σ^2^_C_), and unique-environmental (E) influences (σ^2^_E_). This approach does not take into account the possible existence of genotype — environment interaction: Different genotypes might respond differently to the same environment, or conversely, some genotypes may be more sensitive to changes in the environment than others. Genotype by environment interaction can be assessed in the case that the common and unique environment feature are latent (i.e., unmeasured) variables (i.e., Heath et al., 1989; Heath et al., 1998). This provides a powerful omnibus test to assess whether there is any statistically significant interaction. However, no conclusions can be drawn regarding specific environmental influences. Alternatively, genotype by environment interaction can be detected using one or more measured moderator variable(s), which can make results very informative. For example, the results of one of the first studies that applied this approach suggests that additive genetic influences on depression interact with marital status in women, where genetic influences are more important for unmarried women (Heath et al., 1998).

Having collected one or more moderator variable(s), one can test not only for interaction effects with additive genetic influences (A × M), but also with common environmental influences (C × M) or unique environmental influences (E × M) — that is, moderation of variance components (henceforth referred to as ACE × M). This approach was applied by, among others, Heath et al. (1989), analyzing genetic and environmental variance components on drinking habits as a function of marital status, and Boomsma et al. (1999), analyzing disinhibition as a function of religious upbringing. In the domain of mathematical ability, Tucker-Drob and Harden (2012) tested in a sample of 6,540 4-year-old twin pairs whether the relation between genetic influences in learning motivation and mathematical ability is positively moderated by family socio-economic status (SES), hypothesizing that importance of genetic differences in learning motivation is strengthened among children raised in high SES families but suppressed among children raised in lower SES families: Children with a genetic predisposition to be motivated to learn mathematics, they predicted, may only act upon this predisposition when educational experiences (e.g., books at home) are available — which is more likely to be the case in high SES families. Results suggested that indeed, genetic differences accounted for only a small amount of variation in mathematics scores at low levels of SES, whereas at very high levels of SES, genetic influences accounted for a much larger part. Furthermore, both the main effect of genetic influences on mathematical ability as well as the interaction effect of genetic differences and SES on mathematical ability were completely mediated through genetic factors that influence learning motivation.

Purcell (2002) implemented a structural equation modeling (SEM) parametrization to investigate ACE × M. In the univariate moderation model, interaction effects are modeled directly on the path loadings of the ACE model components. That is, the variances of A, C, and E are fixed to unity, but path coefficients are parametrized as (β_0a_ + β_a_M_ij_), (β_0c_ + β_c_M_ij_), and (β_0e_ + β_e_M_ij_) respectively, where M_ij_ represents a moderator variable for individual *j* from twin family *i*. β_0a_, β_0c_, and β_0e_, are intercepts estimating influences of all components (A, C, and E) independent of *M* and β_a_, β_c_, and β_e_ represent regression coefficients that express the respective interaction effects (A × M, C × M, and E × M).

The aim of the current article was to incorporate a measurement model into the univariate analysis of ACE × M. This is important since statistical findings regarding non-linear effects such as ACE × M interaction effects are dependent on the scale at which the analysis takes place — a simple non-linear transformation can obscure or reveal interaction effects (see e.g., Eaves et al., 1977; Molenaar & Dolan, 2014; Schwabe & van den Berg, 2014; van der Sluis et al., 2012). The incorporation of a measurement model can overcome potential bias due to scale properties (explained in further detail below). For this extension, an alternative ACE × M parametrization was used, which will be introduced below.

## Alternative ACE × M Parametrization

Purcell (2002) estimated ACE × M by moderating the regression of the phenotype on the latent A, C, and E variables such that regression paths have to be squared in order to produce a variance expectation (e.g., σ^2^_A_ = (β_0*a*_ + β_1a_M_*ij*_)^2^ for twin *j* from family *i*). It can, however, be shown that this parametrization is ill determined in the sense that there is no unique maximum likelihood (ML) solution for a given data set. This means that the model is not identified, as it can result in multiple parameter estimates with an equally good fit (see the online supplementary material for a detailed proof). This makes the interpretation of results non-trivial: For instance, confidence intervals (CI) for the moderation effects cannot be interpreted meaningfully nor the sign of the sign of the β_0a_, β_0c_, β_0e_, and β_a_, β_c_, and β_e_, parameters.

Alternatively, we can parametrize ACE × M by specifying the moderator variable to modify the log-transformed variances of A, C, and E (see also Tucker-Drob et al., 2009; Turkheimer & Horn, 2013). In case of a moderator variable that is the same for every twin within a family *i* (e.g., SES), this makes variance components different for families with a different value on the moderator variable. For example, to model C × M, we have for every twin family *i*:
1
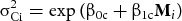

where **M** denotes a vector consisting of the moderator values for every twin family *i* of one moderator variable. β_0c_ represents the intercept (estimating common-environmental variance when **M**_i_ = 0) and β_1c_ represents the C × M interaction effect. Note that in case of a bivariate moderator variable (e.g., high SES = 1, low SES = 0), this results in two possible values for σ^2^_Ci_ (i.e., σ^2^_Ci_ = exp(β_0c_) when **M**_i_ = 0 and σ^2^_Ci_ = exp(β_0c_ + β_1c_), but depending on the distribution of the moderator variable, this can result in a different variance component for every family. The underlying idea behind this parametrization is that a variance cannot be negative. Contrary to Purcell's (2002) parametrization, this parametrization is uniquely identified in that there is only one maximum in the likelihood function. Here, we use this alternative parametrization to integrate a measurement model into the modeling of ACE × M.

## Integration of a Measurement Model

Tests or questionnaires that consist of multiple items may not be equally reliable across the entire range of sum scores — that is, measurement error can be heterogeneous across the trait continuum. For example, cognitive ability tests show little measurement error for average performing participants, but can become less informative for highly able participants as they usually contain only a few very difficult items. Likewise, a depression scale that contains mostly extreme items may be highly informative for depressed participants but less so in the assessment of healthy controls. It has been shown that if phenotypes are heterogeneous across the trait continuum, this can lead to spurious interaction effects in GE interaction models when environmental variables are unmeasured (see Molenaar & Dolan, 2014; Schwabe & van den Berg, 2014). The finding of spurious interaction effects can also be expected in case of ACE × M (see e.g., Tucker-Drob et al., 2009). This can be illustrated by the simple example of genetic influences for IQ being more important in families with a high SES, as described by for example Turkheimer et al. (2003). Assuming that the *true* heritability is constant across SES levels (i.e., β_1a_ = 0) and that there is a ceiling effect in the IQ data in the sense that there is more measurement error at the right tail of IQ scores, then consequently there is less information on high scoring twins and these pairs will seem more alike in a biometric analysis that is not corrected for measurement error. If IQ levels increase with SES, the correlation of the first and second twin of a family will be lower at higher SES levels, leading to higher estimates of genetic variance — and therefore to a positive A × SES interaction effect. Note that this effect is spurious in the sense that they are due to scale properties rather than biological mechanisms.

Schwabe and van den Berg (2014; see also Molenaar & Dolan, 2014) have shown that the incorporation of an item response theory (IRT) measurement model into the biometric analysis can overcome potential bias due to scale properties — results regarding interaction effects are free of artefacts due to heterogeneous measurement error across the trait continuum. Further advantages of the IRT approach include the flexible handling of missing data and the harmonization of traits measured on different measurement scales (see, e.g., van den Berg et al., 2014). For example, when different twin registers have used different IQ tests not comparable in difficulty, IRT can be used to set the items scores on the same scale. The IRT approach is briefly introduced next.

## Item Response Theory

In the IRT framework, a twin's latent trait (e.g., mathematical ability) is estimated based on performance (e.g., on a mathematics test) and on test item properties (e.g., the difficulty of each test item). The simplest IRT model is the Rasch model, also known as the one-parameter logistic model (1PLM), where the probability of a correct answer to item *k* (e.g., of a mathematics test) by twin *j* from family *i*, *p*(*Y_ijk_* = 1), is modeled as a logistic function of the difference between the twin's latent traits score (e.g., mathematical ability) and the difficulty of the item:
2
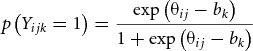

where θ_ij_ refers to the latent trait score of individual twin *j* from family *i*, *b_k_* denotes the difficulty of item *k*, which represents the trait level associated with a 50% chance of endorsing an item. This IRT model is suitable for dichotomous items (e.g., 1 = scored as correct and 0 = incorrect), as is, for example, collected from mathematical ability tests. An underlying assumption of the Rasch model is that all items discriminate equally well between varying abilities: How rapidly the probabilities of a correct answer change with trait level is the same for all items. An extension of the Rasch model, the two-parameter logistic model (2PLM), also estimates discrimination parameters that differ across items. The change in probabilities of a correct answer with trait level can then be different (e.g., faster or slower) among the different items (see e.g., Embretson & Reise, 2009). There are also several IRT models for non-dichotomous data such as ordered categories (e.g., Likert scale data). In this article, the Rasch model was used, but extensions to the 2PLM or ordinal IRT models are straightforward.

### Earlier Research

Tucker-Drob et al. (2009) proposed, next to the biometric ACE × M model, to explicitly model the factor structure of the phenotype at the psychometric level and showed that ignoring non-linearity in the factor structure can lead to the finding of spurious interaction effects, highlighting the need for new methods that integrate a measurement model into the ACE × M biometric model. However, they did not validate their approach using a simulation study, but demonstrated it by reanalyzing ACE × M on IQ data previously analyzed by Turkheimer et al. (2003).

As sum score data were available for 12 separate cognitive tests, they tested whether there was a single factor common to all tests, using Mplus. Subsequently, the ACE × M analysis and a non-linear factor structure were modeled using the Markov chain Monte Carlo (MCMC) estimation program WinBUGS (Lunn et al., 2000), where factor loadings were set to the values retained from the Mplus output.

Also focusing on ACE × M in psychiatric genetics, Eaves (2017) simulated additive and independent genetic and environmental risks for 10,000 monozygotic (MZ) and 10,000 dizygotic (DZ) twin pairs and checklists of clinical symptoms and environmental factors typically found in empirical data. Eaves (2017) showed then that although latent risk scores were analyzed without any interaction effects, sum scores suggested evidence for ACE × M and other effects of modulation and that the integration of an IRT measurement model prevented this bias. Different from the approach we take, however, Eaves (2017) used the non-identifiable solution introduced by Purcell (2002).

Although the simulation study performed by Eaves (2017) captures the essence of the problem, the item-level data were analyzed such that item difficulty parameters were the same for every item. It remains to be established how this solution performs in case of the alternative ACE × M parametrization as described above and in case of different item difficulty parameters. It also remains unclear how much psychometric information (i.e., how many items) is needed to prevent the spurious finding of ACE × M.

Here, we introduce an MCMC method that fits the biometric ACE × M model and the IRT model simultaneously, taking full advantage of the IRT approach. A simulation study was conducted to show that in case of heterogeneous measurement error, the sum score based approach results in the finding of spurious interaction effects while the approach introduced here is unbiased. In a second simulation study, the impact of the psychometric information was investigated by varying the number of items (20, 100, and 250 items) while keeping the number of twin pairs constant.

Next, we applied the new methodology to the data of Dutch twin pairs on mathematical ability and estimated genetic and non-genetic variance components in 12-year-olds for mathematical ability as a function of a family's SES.

### Biometric and Psychometric Model

The full model, consisting of a biometric and psychometric part, is presented in more detail for MZ and DZ twins separately.

### MZ Twins

For each twin pair *i*, the effect of common environmental influences is perfectly correlated within the pair and normally distributed with an expected value consisting of the phenotypic population mean, μ, and the main effect of the moderator variable **M**. Familial influences *F*, consisting of additive genetic influences (A) and common environment (C), were assumed to be normally distributed for every family *i*:
3
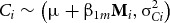

4
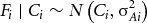

Where μ represents the phenotypic population mean and β_1_*_m_* represents a regression coefficient that expresses the estimated main effect of the moderator variable. In order to model A × M and C × M, for every twin MZ family *i*, variance components were divided into an intercept (representing variance components when **M***_i_* = 0) and a linear interaction term (denoting A × M and C × M respectively):
5
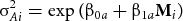

6
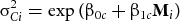

Where β_0_*_c_* (β_0_*_a_*) represents common-environmental (additive genetic) variance when **M***_i_* = 0 and β_1_*_c_* band β_1_*_a_* represent A × M and C × M, respectively.

The expected value of the phenotypic trait, θ*_ij_* of individual twin *j* from family *i* then consisted of the familial effect:
7
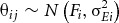


In order to model E × M, variance due to unique-environmental influences, σ^2^_*E*_, was different for every MZ family *i* with a different value on the moderator variable and divided into an intercept (unique-environmental variance when **M***_i_* = 0) and an interaction term:
8
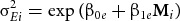


In the psychometric part of the model, the probabilities for correct item response *k* of twin *j* from family *i*, *P_ijk_*, were then modeled conditional on θ*_ij_* in the Rasch model:
9
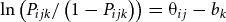

10
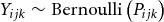

where *b_k_* refers to the difficulty parameter of item *k*. In the simulation studies, it was assumed that item parameters were known.

### DZ Twins

Similar to MZ twin pairs, a normal distribution was used to model a common environmental effect for every DZ family *i* that is perfectly correlated within a twin pair with an expected value consisting of the phenotypic mean and the main effect of the moderator variable **M**:
11
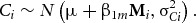


Similar to MZ twin pairs, variance due to common-environmental influences was divided into an intercept and a part that estimates C × M (σ^2^_*Ci*_ = exp(β_0*c*_ + β_1*c*_**M**_*i*_), (see Equation 6).

While the total genetic variance is assumed to be the same for DZ and MZ twins, the genetic covariance in MZ twins is twice as large as in DZ twins, as DZ twin pairs share on average only 50% of their genomic sequence. To model a genetic correlation of 0.5 for DZ twins, first a standard normal distributed additive genetic value was assumed for each DZ family *i*. Then, for each individual twin *j* from family *i*, a normally distributed additive genetic value was assumed, representing the Mendelian sampling term:
12
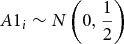

13
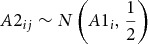


The genetic value was scaled by multiplying it with the standard deviation σ*_Ai_*, where σ^2^_*Ai*_ = exp(β_0*a*_ + β_1*a*_**M**_*i*_). This yielded a genetic effect *A*3*_ij_* that was unique for every individual twin *j* from DZ family *i*:
14
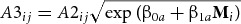


The expectation of the phenotypic variable, θ*_ij_*, then consisted of the common environmental effect for every DZ family *i* and the additive genetic effect for every individual twin *j*:
15
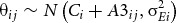

where, as for MZ families, σ^2^_*Ei*_ was divided into an intercept (representing unique environmental variance when **M***_i_* = 0) and an interaction term (σ^2^_*Ei*_ = exp(β_0*e*_ + β_1*e*_**M**_*i*_; see also Equation 8). In the psychometric part of the model, a Rasch model was applied (see Equations 9 and 10) of which the item parameters were assumed to be known.

### Estimation of the Model

We used Bayesian statistical modeling to simultaneously estimate the psychometric and the biometric model. In the Bayesian framework, statistical inference is based on the so-called joint posterior density of all model parameters. The posterior density is proportional to the product of the likelihood function and a prior probability density for unknown parameters (for an introduction to Bayesian statistics see, e.g., Bolstad, 2007). For a detailed description of the estimation procedure and the prior probability densities applied here, the interested reader is referred to the online supplementary material.

### Simulation Study 1

**FIGURE 1:**
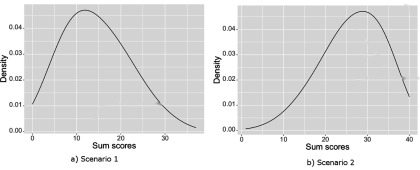
Distribution of the sum scores of the DZ twins as simulated in the simulation study.

The Rasch model was used to simulate responses to 40 phenotypic dichotomous items where item difficulty parameters were assumed known. Two different scenarios were simulated: To mimic a slight floor effect for the distribution of sum scores, in the first scenario, item parameters were simulated from a normal distribution with a mean of 1 and a standard deviation of 1. In the second scenario, item parameters were simulated from a normal distribution with a mean of −1 and a standard deviation of 1 to simulate a slight ceiling effect. To give an idea of the severity of the skewness, the distributions of the simulated sum scores of all DZ twins are displayed in Figure 1 for both the floor effect scenario (left) and the ceiling scenario (right). The three different methods for estimating skewness proposed by Joanes and Gill (1998) resulted in values in the range 0.7223977, 0.7231508 in the first scenario and −0.5260599, −0.5266083 in the second scenario.

In each scenario, 250 datasets were simulated, each consisting of 2,000 twin pairs where the percentage of MZ twins was fixed to 28% of all pairs. The phenotypic population mean, μ, was fixed to 0. Using similar values as Purcell (2002), *exp*(β_0_*_a_*) was set to 0.25, *exp*(β_0_*_c_*) was 0.25 and *exp*(β_0_*_e_*) was fixed to 0.5. All interaction effects (β_1_*_a_*, β_1_*_c_*, and β_1_*_e_*) were set to zero. For every MZ and DZ family, a dichotomous moderator value was simulated such that the moderator explained approximately 11% of the total phenotypic variance (with β_1_*_m_* fixed to 0.7). The simulated datasets were then analyzed using the above described method and a sum score approach. In the sum score approach, all scores were divided by the standard deviation of the sum score of those twins who scored 1 on the moderator value in order to set both the IRT and sum score analysis on the same scale and make results comparable. To estimate the power of both models, the 95% highest posterior density interval (HPD, see e.g., Box & Tiao, 1972) was determined for each parameter and the percentage of simulated datasets in which this interval did not contain zero was calculated. Note that a power higher than 0.05 for one of the interaction effects (e.g., β_1_*_a_*, β_1_*_c_*, and β_1_*_e_*) suggests an increased (i.e., more than expected due to chance) false positive rate.

### Simulation Study 2

In an additional simulation study that is described in the online supplementary material, the impact of the psychometric information (e.g., the influence of the number of items on parameter estimates) was investigated by varying the number of items (20, 100, and 250) while fixing the number of twin pairs at 1,000. Details and results of this simulation study can be seen in the online supplementary material.

All simulations were carried out using the software package R (R Development Core Team, 2008). As an interface from R to JAGS, the R package rjags was used (Plummer, 2013). After an adaption phase of 5,000 iterations and a burn-in phase of 50,000 iterations, the characterization of the posterior distribution for the model parameters was based on an additional 25,000 iterations from 1 Markov chain.

## Results: Simulation Studies

The true parameter values, posterior means and the means of posterior standard deviations, averaged over 250 replications, can be found in Table 1 for the first scenario from simulation study 1. The estimated power for each parameter under both models can be found in Table 2. All power estimates for β_1_*_m_*, *exp*(β_0_*_a_*), *exp*(β_0_*_c_*), and *exp*(β_0_*_e_*) were equal to 1 under both models and are therefore not tabulated here.

**TABLE 1:** Posterior Means (*SD*) Averaged Over 250 Replications

	β_1_*_m_*	*exp*(β_0_*_a_*)	*exp*(β_0_*_c_*)	*exp*(β_0_*_e_*)	β_1_*_a_*	β_1_*_c_*	β_1_*_e_*
True value	0.70	0.25	0.25	0.50	0.00	0.00	0.00
Sum scores	0.60 (0.03)	0.17 (0.05)	0.17 (0.04)	0.50 (0.02)	−0.33 (0.81)	−0.31 (0.56)	−0.24 (0.10)
	0.02	0.05	0.04	0.02	0.83	0.50	0.08
IRT	0.70 (0.03)	0.22 (0.07)	0.24 (0.05)	0.51 (0.03)	-0.07 (0.80)	0.00 (0.57)	0.01 (0.11)
	0.03	0.07	0.05	0.03	0.86	0.51	0.11

Second line: Mean of posterior standard deviations.

**TABLE 2:** Power to Find Interaction Effects for Both Models, Defined as the Number of Simulated Datasets in Which the 95% HPD Interval Did Not Contain Zero

	β_1_*_a_*	β_1_*_c_*	β_1_*_e_*
Sum scores	0.12	0.17	0.81
IRT	0.06	0.05	0.05

In the first scenario, a negatively skewed sum score distributed was mimicked, resulting in a slight ceiling effect, which resulted in biased parameter estimates: The main effect of the moderator variable, β_1_*_m_* as well as *exp*(β_0_*_a_*) and *exp*(β_0_*_c_*) were underestimated. More importantly, the analysis resulted in relatively high interaction parameters (i.e., β_1_*_a_*, β_1_*_c_* and β_1_*_e_*). Table 2 shows that, in particular, spurious findings of E × M interactions can be expected when the sum score approach is used. When the IRT approach was used, parameter estimates were much closer to their true values, with only a slight bias in *exp*(β_0_*_a_*) and *exp*(β_1_*_e_*). Power estimates for the IRT model were either equal to or only slightly above (0.06 for β_1_*_a_*) the 5% that can be expected when an interaction would be found simply due to chance. In other words, the IRT approach did not result in the finding of spurious interaction effects.

In the second scenario, a slight floor effect was mimicked, resulting in a positively skewed sum score distribution. Since the second scenario was the mirror image of the first scenario, parameter estimates were very similar to those found in the first scenario with spurious interaction effects in the opposite direction (i.e., β_1_*_a_* = 0.36[0.85], β_1_*_c_* = 0.29[0.67], and β_1_*_e_* = 0.24[0.10]). To save space, the results of the second scenario are not tabulated but can be obtained from the first author.

The results of the additional simulation study (see the online supplementary material for details) indicate that there was only a small decrease in standard deviations and standard errors with increasing number of items. Also, the increase in precision with increasing sample size was small, suggesting that as much as 20 items are sufficient to fit the ACE × M model.

### Application to Mathematical Ability

The model described above was used to investigate ACE × SES in mathematical ability of 2,110 12-year-old Dutch twin pairs. Mathematical achievement was measured with the Eindtoets Basisonderwijs, which is a Dutch national achievement test that is administered in the last year of primary education. The Eindtoets Basisonderwijs test assesses what a child has learned during primary education and consists of 290 multiple choice items in four different subjects (language, arithmetic/mathematics, study skills, and world orientation [optional]). Here, we used the 60 dichotomous item scores (0 = *incorrec*t, 1 = *correct*) of the mathematics subscale of this test.

### Data

The data of 12-year-old twins from the Netherlands Twin Register from birth cohorts 1998–2000 were used to link individual twins to their dichotomous item scores (coded as 0 = *incorrect*, 1 = *correct*) on the mathematics subscale of the Eindtoets Basisonderwijs test. The linking procedure and harmonization of different test versions is described in detail in Schwabe et al. (2017). This led to a dataset for a total of 4,220 twins, forming 2,110 twin pairs of which 581 were MZ and 1,529 DZ pairs. Of the MZ twin pairs, 299 were female and 282 pairs were male. The DZ pairs consisted of 360 male, 309 female, and 860 opposite-sex pairs. For 711 individual twins, item scores were unknown. The reasons that the scores were missing were either that the child had not reached final grade yet (*n* twins = 52), the child was attending special education (*n* twins = 34), a different test was used at the school the twin was attending (*n* twins = 13), the child (*n* twins = 2), or the whole school (*n* twins = 1) did not attend the test, or the reason was unknown (*n* twins = 609).

### Family SES

The NTR collects longitudinal data from all registered twins by mailed surveys. Among other information, parents are asked for the family SES measured as highest parental occupation at ages 3, 7, and 10 of their twins. Family SES was scored in five different categories that approximately translate to: (1) ‘Unskilled labor’, (2) ‘Job for which lower vocational education is required’, (3) ‘Job at medium level’, (4) ‘Job at college level’, and (5) ‘Job at university level’. In order to gain statistical power, the data of all ages were combined into one measure, meaning that when the value was missing at age 10, the missing value was substituted with the value measured at age 7 or if this value was missing, with the value measured at age 3. The lowest correlation between multiple SES scores was 0.72 between SES at age 3 and SES at age 10. Family SES scores were available for a total of 1,708 families (81%), with SES scores of 334 families (16%) measured at age 10, scores of 831 (39%) families at age 7, and of 543 (26%) families at age 3.

### Analysis

For an easier interpretation, SES categories were summarized in one dichotomous dummy variable, coded as 0 (i.e., families with a score of or lower than 3 on family SES) and 1 (i.e., families with a score of or higher than 5 on parental SES). We interpret these two categories as ‘families with average or low SES’ (coded as 0) and ‘families with high SES’ (coded as 1). Seven hundred and twenty (34%) families had a high family SES and 988 (47%) an average or low SES (see also Schwabe et al., 2017). Details on the distribution of the SES moderator can be found in Table 3.

**TABLE 3:** Total Number (Percentage) of Twin Pairs With High SES, Average or Low SES, or Missing Data

Sample	High SES	Average or low SES	Missing SES
MZ twin pairs	202 (35%)	282 (48%)	97 (17%)
DZ twin pairs	518 (34%)	706 (46%)	305 (20%)
All pairs (MZ + DZ)	720 (34%)	988 (47%)	402 (19%)

As a measurement model for the mathematics item scores, the 1PLM (Verhelst et al., 1995) version of an IRT model was used. In the OPLM, item difficulty parameters, *b_k_*, are estimated and item discrimination parameters, *a_k_* are imputed as known constants. Item parameters were estimated by the psychometric group at the testing company Cito and imputed as known parameters in the analysis (for more details, see Schwabe et al., 2017).

As there were twins with missing SES data, independent Bernoulli distributed prior distributions were defined (SES*_i_* ~ Bernoulli[π]). On the probability π (i.e., the probability that the dichotomous SES moderator takes the value 1), a beta-distributed hyperprior was used, which was different for MZ and DZ twins (π*_mz_ ~* Beta[1,1] and π*_dz_ ~* Beta[1,1]). Note that by using prior distributions, all available data could be analyzed without missing SES values resulting in a reduction of sample size.

After a burn-in phase of 50,000 iterations, the characterization of the posterior distribution for the model parameters was based on a total of 175,000 iterations from five different Markov chains. The posterior means and standard deviations were calculated for each parameter, as was the 95% HPD interval (see e.g., Box & Tiao, 1972). The HPD can be interpreted as the Bayesian analogue of a CI. When the HPD does not contain zero, the influence of a parameter can be regarded as significant. This, however, does not hold for the variance components of this particular application, as these are bounded at zero, because the discrimination parameters for the OPLM were quite high (in the range of [1:9]), which resulted in a very low phenotypic variance.

## Results: Application

**FIGURE 2:**
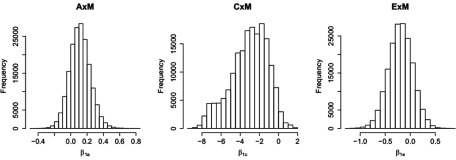
Application: Moderating effects of a family's SES on individual differences in mathematical ability. Histograms of the posterior distribution of β_1_*_a_* (A × SES, left), β_1_*_c_* (C × SES, middle), and β_1_*_e_* (E × SES).

The posterior means for the intercepts, interaction effects and estimated heritability, *h*^2^, can be found in Table 4. Histograms of the posterior distributions of all interaction effects can be seen in Figure 2.

**TABLE 4:** Posterior Means (*SD*) of Parameters

	Posterior point estimate (*SD*)	95% HPD
β_1_*_m_*	0.1078 (0.0126)	[0.0833; 0.1329]
*exp*(β_0_*_a_*)	0.0511 (0.0048)	[0.0410; 0.0598]
*exp*(β_0_*_c_*)	0.0051 (0.0029)	[0.0008; 0.0107]
*exp*(β_0_*_e_*)	0.0159 (0.0023)	[0.0117; 0.0204]
β_1_*_a_*	0.1,132 (0.1,263)	[−0.1,364; 0.3,627]
β_1_*_c_*	−3.0777 (1.9,280)	[−7.3,886; −0.0300]
β_1_*_e_*	−0.1,958 (0.2,426)	[−0.6,769; 0.2,759]
*h*^2^	0.7,088 (0.0565)	[0.5,800; 0.7,995]

HPD = highest posterior density interval.

Heritability was defined as 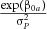, where σ^2^_*P*_ = exp(β_0*a*_) + exp(β_0*c*_) + exp(β_0*e*_). The results suggest that the largest part of phenotypic variance could be explained by genetic influences, a substantial part by unique environmental influences and a negligibly small part by common environmental influences. A significant and negative C × SES interaction effect was found, meaning that common environmental influences are less important in creating individual differences in mathematical ability in families with a high SES than in creating individual differences in mathematical ability in twin pairs with a low or average SES.

## Discussion

In this paper, the biometric modeling of moderation of variance decomposition (i.e., ACE × M) was extended with an IRT model at the phenotypic level, making it possible to analyze item data rather than sum scores. We applied the extended model to data on mathematical ability.

We modeled ACE × M on the log-transformed variances, using a parametrization that is uniquely identified and therefore to be preferred over the parametrization by Purcell (2002). Note, however, that this parametrization is also limited in the sense that, while the parametrization of Purcell (2002) results in a parabolic form of interaction effects, the alternative parametrization is either monotically increasing or decreasing with respect to the moderator. Although this might lead to an easier (biological) interpretation (see also Turkheimer & Horn, 2013), it can also result in bias when the true moderation pattern is parabolic: Imagine, for example, that the importance of genetic influences in explaining variation in mathematical ability is moderated by study motivation in the sense that heritability is high only in children with a really low or really high study motivation. In this hypothetical example, the log-transformation parameterization would, depending on the distribution of the empirical data, not detect any interaction effects and therefore lead to wrong conclusions.

To show that in the case of heterogeneous measurement error, the sum score approach results in spurious interaction effects while the proposed method is unbiased, two scenarios were simulated. While the first scenario resulted in a situation often encountered in cognitive ability studies with gifted children (i.e., a ceiling effect), the second scenario is typical for psychopathology studies (i.e., a floor effect by having many extreme items that are not endorsed by healthy controls). The results showed that spurious interaction effects can be expected when the sum score approach is used, in particular in the case of an E × M interaction. On the other hand, the IRT approach was unbiased. A second simulation study suggested that 20 items are sufficient to use the IRT method.

The method was applied to investigate the moderating influence of SES on variance components in mathematical ability of 2,110 12-year-old Dutch twin pairs. The results suggest that most of the phenotypic variance could be explained by genetic influences, resulting in a relatively high heritability of 71%. A substantial part of the variance in mathematical ability could be explained by unique environmental influences and a small part by common environmental influences. Furthermore, a negative C × SES interaction effect was found, meaning that common environmental influences were less important in creating individual differences in mathematical ability in families with a high SES than in creating individual differences in mathematical ability in twin pairs with a low or average SES. Children from low-SES families are generally disadvantaged. For example, they live in disadvantaged neighborhoods with poor facilities and inferior schools or have fewer books at home (see, e.g., Evans, 2004). These disadvantages, captured in the C component, seem to contribute to the observed greater variation in mathematical ability of children from low-SES families. An implication of this result might be that family-based environmental interventions directed at enhancing mathematical ability are more effective in lower SES families (see also Hanscombe et al., 2012, for a similar argument in the context of IQ). We have, however, to be cautious in drawing conclusions: the methodology introduced here is concerned with the moderation of variance components unique to the phenotypic variable. That is, phenotypic scores are corrected for the influence of SES and, consequently, any genetic or environmental effects that operate through or are common with SES are partialled out. This makes interpretation difficult, because SES has consistently been associated with higher academic achievement and cognitive performance throughout childhood and adolescence (see, e.g., Sirin, 2005; White, 1982) suggesting a (genetic) correlation between SES and mathematical ability. This correlation might result in an underestimation of the mathematical ability of twins from high SES families, because their phenotypic latent scores are corrected for a measure that actually correlates with the trait of interest. To gain more confidence in the presented results, in future research, the analysis should be replicated by applying the bivariate moderation model (see, e.g., Purcell, 2002) in which interaction effects are considered not only on influences unique to the phenotype but also on influences that are common to the phenotype and the moderator variable.

Our findings are contrary to earlier research conducted by Tucker-Drob and Harden (2012), who found no moderating effects of SES on common environmental variance, but rather that SES influences the extent to which additive genetic factors contribute to individual differences in mathematical ability. This inconsistency in findings might be due to age differences (i.e., a sample of 12-year-olds compared to a sample of 4-year-olds), the different statistical assumption that were made (i.e., here we analyzed moderation on latent traits, whereas Tucker-Drob & Harden, 2012, used sum scores) or the fact that the samples originated from different countries (i.e., the Netherlands and the United States) in which SES might be more or less a factor in children's mathematical ability.

In the method described, an IRT model was used to model ACE × M at the latent phenotypic level such that trait scores are corrected for measurement error. However, measurement error might not only appear at the level of the phenotype but also in the measurement of the moderator variable. To gather information on the environment of a family or an individual twin, often self-reports (e.g., questionnaires) are used. To correct for measurement error in the moderator level, in future research, the method will be extended to include an IRT model also at the level of the moderator variable. In case of heterogeneous measurement error in the moderator variable, it is to be expected that this to be developed method performs better than the method introduced here (e.g., using an IRT model only at the phenotypic level), but it is unclear how large the differences in the two methods are and whether heterogeneous measurement error in the moderator variables also results in the spurious finding of interaction effects. Heterogeneous measurement error in the moderator variable can, for example, occur when the moderator resembles a psychological trait. Assume, for example, that one is interested in the influences of study motivation on the importance of genetic, common environmental, and unique environmental influences on individual differences in educational achievement: A scale that measures study motivation might be very reliable for students with an average motivation, but it may be very difficult to distinguish the motivated students from the very motivated students (i.e., the measurement error is higher at the upper tail of the distribution).

A drawback of the method is that it is computationally intensive and, depending on the number of items and twin pairs, can take several hours to complete. In future research, more efficient sampling algorithms will be applied to lighten the computation burden. The model can furthermore be expanded to include both linear and quadratic interactions on the paths (such that the ACE variance estimates are quadratic with respect to the moderator). Therefore, one can test whether genetic variance can be modeled as an inverted U-shaped curve, with the highest genetic variance in the ‘average’ environment. In this study, we considered only linear interaction effects. It has been shown that the integration of a curvilinear interaction effects is problematic even in the case that environmental measures are unmeasured (Schwabe & van den Berg, 2014). Furthermore, the additional parameters are likely to require larger sample sizes than the ones considered here.

## Supplementary material

To view supplementary material for this article, please visit https://doi.org/10.1017/thg.2017.59
